# Case of Forced Twin Labor at Seven Months

**Published:** 1881-12

**Authors:** L. E. Ellis


### Case of Forced Twin Labor at Seven Months.

Dr. L. E. Ellis.

Mrs. C., age 34, of German birth, called me in on the morning of October 12, at 12.15 A. M., and I found her in labor and with a severe chill. She said the waters had broken at 12 o'clock, and the bed and her clothing were completely wet through; said about a month before she had flowed considerably, the discharge being about half water and half blood, but she thought nothing of it, and kept at work saying nothing to any one until the day before she called me. As she was cleaning the house, and standing upon a chair, reaching up to clean the doors and windows, she noticed she "flowed" so as to be wet through. She then told her husband, who advised her to stop work and lie down, which was done. She had some light pains, but they all passed away in an hour or two, and she resumed work. In the evening she felt quite weak again, but soon went to sleep, being often awakened on account of fullness in the abdomen. This interfered with her at intervals, after which it lessened somewhat, and she again fell asleep. She had been somewhat similarly affected throughout this pregnancy.

I made an examination, and found the os well dilated and soft, and the head descending into the pelvis, but since the waters had escaped, she had felt no pains. At this time I gave her a drachm of ergot-Squibbs, Fl. extract, and after waiting fifteen minutes gave a drachm more, made friction over the womb, dilated the os with my hand, and still there was no pain. I therefore gave more ergot and waited further developments until six A. M., when I called counsel. This physician made an examination and advised the repetition of the ergot and dilation of the os, and still there was no pain. I now made pressure over the womb and the child descended without any trouble and was delivered. We waited a short time to see if she would not have pains to deliver the second, but as none occurred we made pressure in the same manner, and the second was born dead. The first was alive, but very weak, and only lived thirty-six hours. This one weighed four pounds, the second one, three and one-half pounds.

